# Deep learning-assisted diagnosis of chronic atrophic gastritis in endoscopy

**DOI:** 10.3389/fonc.2023.1122247

**Published:** 2023-03-06

**Authors:** Yanting Shi, Ning Wei, Kunhong Wang, Jingjing Wu, Tao Tao, Na Li, Bing Lv

**Affiliations:** ^1^ Department of Gastroenterology, Zibo Central Hospital, Zibo, Shandong, China; ^2^ Department of Internal Medicine, Zhangdian Maternal and Child Health Care Hospital, Zibo, Shandong, China; ^3^ School of Computer Science and Technology, Shandong University of Technology, Zibo, Shandong, China

**Keywords:** endoscopy, gastric cancer, transfer learning, deep learning - artificial intelligence, chronic atrophic gastritis (CAG)

## Abstract

**Background:**

Chronic atrophic gastritis (CAG) is a precancerous condition. It is not easy to detect CAG in endoscopy. Improving the detection rate of CAG under endoscopy is essential to reduce or interrupt the occurrence of gastric cancer. This study aimed to construct a deep learning (DL) model for CAG recognition based on endoscopic images to improve the CAG detection rate during endoscopy.

**Methods:**

We collected 10,961 endoscopic images and 118 video clips from 4,050 patients. For model training and testing, we divided them into two groups based on the pathological results: CAG and chronic non-atrophic gastritis (CNAG). We compared the performance of four state-of-the-art (SOTA) DL networks for CAG recognition and selected one of them for further improvement. The improved network was called GAM-EfficientNet. Finally, we compared GAM-EfficientNet with three endoscopists and analyzed the decision basis of the network in the form of heatmaps.

**Results:**

After fine-tuning and transfer learning, the sensitivity, specificity, and accuracy of GAM-EfficientNet reached 93%, 94%, and 93.5% in the external test set and 96.23%, 89.23%, and 92.37% in the video test set, respectively, which were higher than those of the three endoscopists.

**Conclusions:**

The CAG recognition model based on deep learning has high sensitivity and accuracy, and its performance is higher than that of endoscopists.

## 1 Introduction

According to the latest World Cancer Report released by International Agency for Research on Cancer (IARC), 1,089,103 new cases of gastric cancer and 768,793 deaths were reported worldwide in 2020, accounting for 5.6% and 7.7% of total cancer incidence and deaths, ranking fifth and fourth respectively (1). Most gastric cancers are gastric adenocarcinomas and CAG is the most common stage of progression to gastric adenocarcinoma (2, 3). Studies have shown that the 5-year incidence of gastric cancer in patients with CAG is 1.9% (3). Some studies in China and Japan have shown that the prevalence of CAG is higher than 50% (4–6).

The collection of biopsies during gastroscopy and pathological analysis is the “gold standard” for diagnosing CAG. This depends significantly on the endoscopist’s ability to collect biopsies (7). Studies have shown that CAG can be detected by white-light endoscopy but with poor accuracy (3). The sensitivity of endoscopic diagnosis of atrophic gastritis is 61.5% in the antrum and 46.8% in the body of the stomach (8). The manual operation of physicians to identify lesions with the naked eye renders it difficult to exclude missed diagnoses owing to fatigue and inexperience. Therefore, seeking an objective and accurate method to identify CAG is very important to slow down or interrupt gastric cancer progression and reduce endoscopists’ workload.

In recent years, artificial intelligence (AI) techniques, represented by deep learning (DL), have been widely used in various medical imaging fields. Examples include disease detection (9, 10), disease prediction (11, 12), and organ detection (13, 14). AI techniques have also shown excellent performance for the diagnosis of digestive diseases. Ueyama et al. (15) constructed a DL computer-aided diagnosis system based on narrow-band imaging to diagnose early gastric cancer, and the accuracy and sensitivity were 98.7% and 98%, respectively. Shichijo et al. (16) constructed a convolutional neural network (CNN) (17, 18) and evaluated its ability to diagnose Helicobacter pylori infection, and the accuracy and sensitivity were 87.7% and 88.9%, respectively. Zhao et al. (19) developed a DL-based assisted diagnostic system for the localization of colon polyps, with a sensitivity of 98.4% in prospective validation. AI also showed excellent ability in CAG diagnosis. For example, Guimarães et al. (20) and Mu et al. (21) automatically extracted endoscopic image features to identify CAG by DL techniques with an accuracy of 93% and 95%, respectively. CAG is endoscopically visible as a red-white mucosal, predominantly white, exposed section of the mucosal blood vessels, and it can be accompanied by mucosal granules or nodules (22, 23). We aim to capture these visible or subvisible image features for CAG recognition using a new DL model, providing more evidence for the feasibility of AI-aided diagnosis of CAG.

CNN is the mainstream algorithm used in the field of image recognition. Since 2017, transformers (24) have shown powerful capabilities in image classification (25, 26), semantic segmentation (27, 28), and object detection (29, 30). In this study, we selected four SOTA DL networks for comparison: two CNNs and two transformers. A new CAG recognition model was constructed based on one of them. The results showed that the model’s accuracy, sensitivity, and specificity were better than those of endoscopic experts.

## 2 Materials and methods

### 2.1 Data collection and preprocessing

In this study, three datasets were collected: (1) an internal image dataset from Zibo Central Hospital, which was used for the training and internal testing of the model; (2) an external test set, an image dataset from the Zhangdian Maternal and Child Health Care Hospital; and (3) video test set, a video dataset from Zibo Central Hospital. The pathology results support all images and videos. Images and videos from Zibo Central Hospital were captured using an Olympus GIF-HQ290 or GIF-H290Z (Olympus, Tokyo, Japan). Images from Zhangdian Maternal and Child Health Care Hospital were captured by Pentax EG29-i10 (Pentax, Tokyo, Japan). All images and videos were captured in the normal white imaging mode. The resolution of the original image was 1920 × 1080 pixels and the format was BMP. The resolution of the original video was 1920 × 1080 pixels, the encoding method was MJPEG, and the frame rate was 25 frames per second (fps). We labeled each image as CAG or CNAG. For videos, one label per video was equivalent to patient-specific labeling.

Internal image dataset: We reviewed the images of patients who underwent gastroscopy at Zibo Central Hospital between June 2020 and June 2022 and had pathological results of chronic gastritis. To reduce interference, we excluded images based on the following conditions: poor quality, inadequate preparation of the digestive tract, altered gastric anatomy because of gastric surgery, and other diseases. The final dataset included 10,361 images from 3,718 patients. Based on the pathology results, we divided the dataset into two categories, CAG and CNAG. The CAG dataset contained images of 1933 patients with CAG, 1114 men and 819 women, with a mean age of 57.15 (± 10.49), 921 with mild atrophy, 984 with moderate atrophy, and 28 with severe atrophy. The CNAG dataset contained images of 1,785 patients with CNAG, 883 men and 902 women, with a mean age of 43.9 (± 13.23). A total of 5219 CAG images were obtained, including 3,826 images of the gastric sinuses, 1184 images of the gastric horns, and 209 images of the gastric body. A total of 5,142 CNAG images were obtained, including 3,545 images of the gastric sinuses, 980 images of the gastric horns, and 617 images of the gastric body.

External test set: We reviewed images of patients who underwent gastroscopy at Zhangdian Maternal and Child Health Care Hospital between January 2022 and October 2022 and had pathological results of chronic gastritis. The inclusion and exclusion processes were identical for the internal image dataset. Finally, 300 images from 116 patients with CAG and 300 images from 98 patients with CNAG were included.

Video test set: We collected video clips of patients who underwent gastroscopy at the Zibo Central Hospital between September 2022 and October 2022. The videos were deliberate scans of the entire gastric region performed by the endoscopist during endoscopy. The exclusion criteria were the same as those used for the internal image dataset. Fifty-three patients with CAG and 65 patients with CNAG were included in the study. The mean duration of the 118 video clips was 50.16 ± 9.57 seconds.

**Figure 1:**
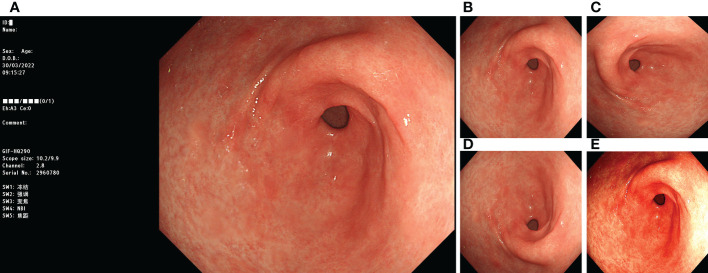
Image preprocessing. **(A)** original image; **(B)** invalid area removed, resize to 512*512 pixels; **(C)** rotate 90°counterclockwise; **(D)** flip vertically; **(E)** color random dither.

Before training with the DL model, we processed the images involved in the training. First, we removed the invalid parts of the image and scaled the image to 512 × 512 pixels. We adopted image-enhancement strategies during model training, such as random rotation, flipping, and color dithering, to improve the model’s generalisation ability. The processing method is illustrated in **Figure 1**.

This study was approved by the ethics committees of the two hospitals involved (No. 202201016, Zibo Central Hospital. No. 202210019, Zhangdian Maternal and Child Health Care Hospital). The patients in the video test set provided written informed consent prior to participation. The ethics committee waived the requirement for informed consent for patients involved in retrospective imaging.

### 2.2 Deep learning method

The experimental hardware environment included an Intel i9 12900 K CPU, an Nvidia GeForce GTX 3090 GPU, and 32 GB of RAM. The experimental software environment included Ubuntu 22.04, CUDA 11.3, Anaconda 4.14, and PyTorch 1.12.1.

**Figure 2:**
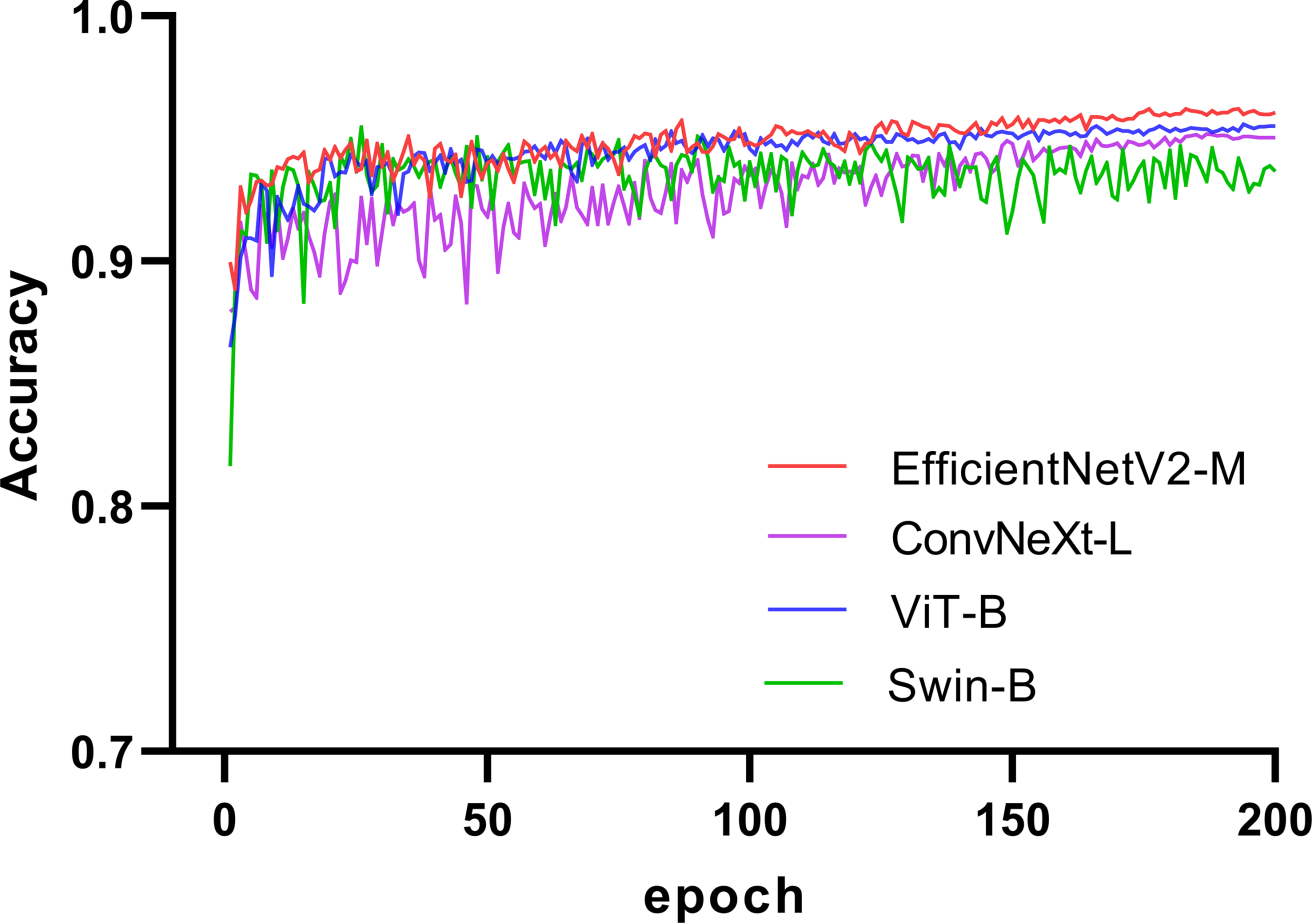
Training process of the four networks.

We selected four SOTA DL networks for inclusion in this study: EfficientNetV2 (31), ConvNeXt (32), ViT (25), and Swin (26). Their commonly used versions, EfficientNetV2-M (31), ConvNeXt-L (32), ViT-B (25), and Swin-B (26), were selected based on the computing power of GPU for training. EfficientNetV2 and ConvNeXt are representative CNN. ViT and Swin are representative transformer networks that have emerged in recent years. We used a pretrained model on ImageNet (33, 34) for transfer learning (35, 36). A significant problem in medical image analysis is that the datasets are relatively small, resulting in less-accurate trained models. Thus, transfer learning can effectively solve this problem. In recent years, transfer learning has achieved good results in medical image analysis (37, 38), which can improve the accuracy of models and accelerate training (39, 40). The training process for the four networks is illustrated in **Figure 2**. The accuracy of each network in the validation set increased with an increase in the number of iterations. After 150 epochs, the accuracy of EfficientNetV2-M, ConvNeXt-L, and ViT-B on the validation set stabilized and Swin-B oscillated in an interval. In summary, EfficientNetV2-M outperformed the other three networks; therefore, we selected it for further optimization.

**Figure 3:**
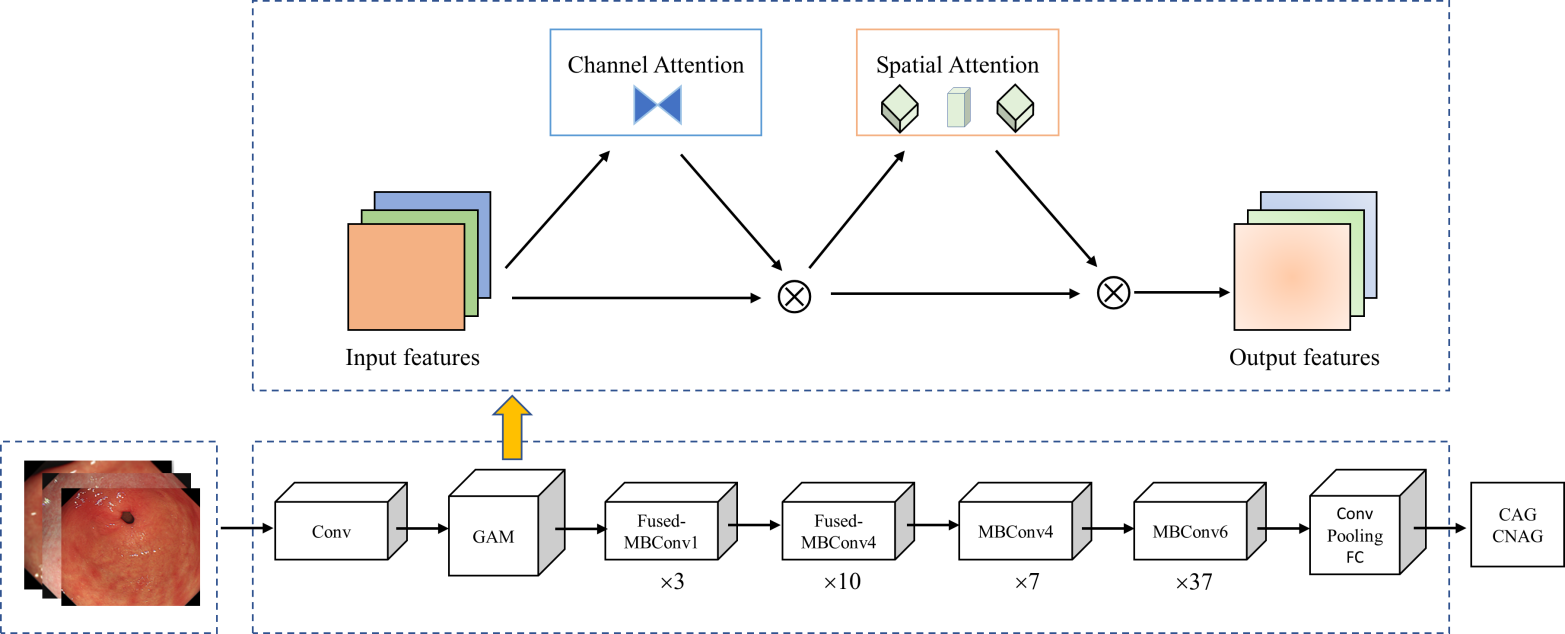
GAM-EfficientNet architecture. Conv represents convolution; Pooling is average pooling layer; FC is full connection layer; Ä represents element-wise multiplication; ×n is repeat times.

**Figure 4:**
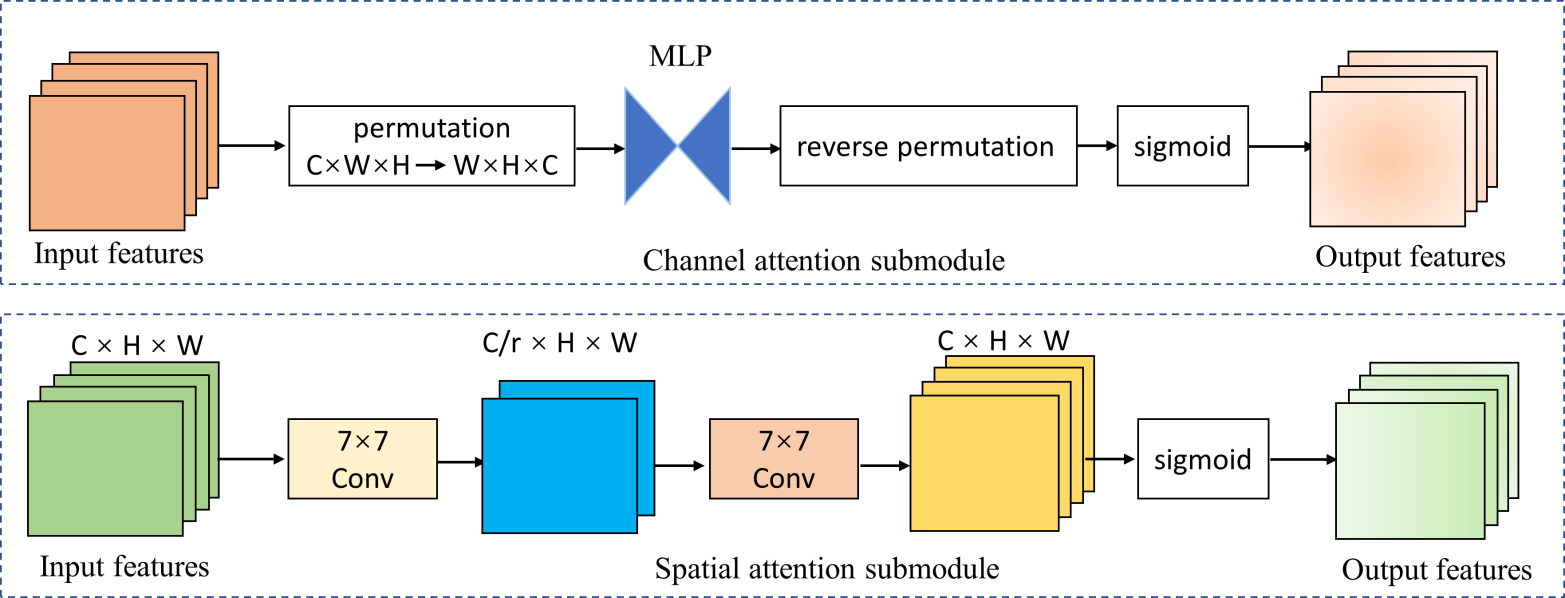
Channel attention and spatial attention submodule. MPL represents multi-layer perceptron; r represents reduction ratio; W, H, and C represent the feature map’s width, height, and number of channels.

We introduced the global attention mechanism (GAM) (41) module based on EfficientNetV2-M to enable the network to focus more on the critical information in the CAG region. The improved network is called GAM-EfficientNet and its structure is shown in **Figure 3**. The GAM module mainly consists of a channel attention submodule (CAM) and spatial attention submodule (SAM), and its structure is shown in **Figure 4**. The CAM first performs dimensional conversion for the input feature map. Following dimensional conversion, the feature map is input into a two-layer multilayer perceptron (MLP). MLP is an encoder-decoder structure that magnifies cross-dimensional channel-spatial dependencies. Subsequently, it is converted to the original dimension and output by sigmoid processing. In SAM, two convolution kernels of 7 × 7 are used for spatial information fusion. GAM amplifies the global dimension-interactive features by adding an element-wise multiplication operation between CAM and SAM.

With other fixed parameters, we selected the optimal parameters of the network using four cross-validations. The following were the final parameters: batch-size was 16, optimizer was AdamW algorithm, initial learning rate was 0.001, learning rate decay strategy was cosine decay (42), and weight decay coefficient was 0.01. After the parameters were determined, the network was retrained to obtain the final CAG recognition model.

### 2.3 Model evaluation

We invited three endoscopists with more than 10 years of experience in endoscopic operations to participate in the test. They diagnosed randomly ordered images/videos in the three test sets without being aware of the pathological findings. The test results were compared with those of GAM-EfficientNet.

The CAG identification in this study was a binary classification problem. We evaluated the model’s performance by calculating the sensitivity (recall), specificity, precision, accuracy, and F1-score, and by plotting the receiver operating characteristic (ROC) curve. The relevant formulas are as follows:


$$Sensitivity=Recall=\frac{TP}{TP+FN}$$

$$Specificity=\frac{TN}{TN+FP}$$

$$Precision=\frac{TP}{TP+FP}$$

$$Accuracy=\frac{TP+FN}{TP+FP+FN+TN}$$

$$F1-score=\frac{2\times Precision\times Recall}{Precision+Recall}$$

where TP, FP, FN and TN represent the numbers of true positives, false positives, false negatives, and true negatives. All statistical analyses were performed using GraphPad Prism 9.3.1 (GraphPad Software, Inc., San Diego, CA, USA).

However, although AI has demonstrated excellent diagnostic performance (43–45), it has a significant drawback that is difficult to explain (46). Explainable AI is an important research direction in medical AI (47). One effective method is to generate heatmaps of the images. We used a gradient-weighted class activation map (Grad-CAM) (46) to create heatmaps showing the regions in which the model predicts the CAG. This can assess whether the model identification process is correct and provide aid to the endoscopist for diagnosis.

## 3 Results

### 3.1 Dataset

A total of 10,961 endoscopic images and 118 video clips from 4,050 patients were included in the study. To train the final model, the internal image dataset was randomly divided into training, validation, and internal test sets at a ratio of 3:1:1. The training and validation sets were used to train the model. The internal test set was not involved in the training and was only used to evaluate the diagnostic capability of the model. The details of the internal image dataset, external test dataset, and video test set are listed in **Table 1**.

**Table 1:** Details of the three datasets.

Category	Internal image dataset				vExternal validation set		Video validation set
	Patients	Trian Set	Validation Set	Test Set	Patients	Image	Patients
CAG	1,933	3,131	1,044	1,044	116	300	53
CNAG	1,785	3,085	1,028	1,029	98	300	65
Total	3,718	6,216	2,072	2,073	214	600	118

### 3.2 Model comparison

We tested five DL models on our internal test set, and the test results are listed in **Table 2**. The main performance metrics of the GAM-EfficientNet are higher than those of the other four networks. The combined performance metric F-Score of GAM-EfficientNet is 94.26%, higher than the second-best performer, EfficientNetV2-M, by 1.4 percentage points. **Supplementary File 1** contains complete training and testing process data for the models.

**Table 2:** Diagnostic performance of five models on the internal test set (n%).

Diagnosed by	Sensitivity	Specificity	Precision	Accuracy	F1-score
ConvNeXt-L	92.91	90.09	90.49	91.50	91.68
ViT-B	93.40	90.38	90.78	91.90	92.07
Swin-B	92.53	89.12	89.61	90.83	91.05
EfficientNetV2-M	92.91	92.71	92.82	92.81	92.87
GAM-EfficientNet	94.35	94.07	94.17	94.21	94.26

### 3.3 Performance evaluation

**Figure 5:**
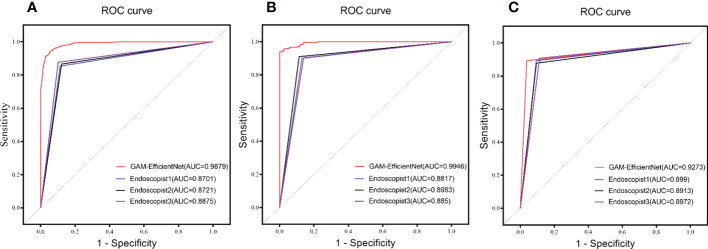
ROC Curve of GAM-EfficientNet and endoscopists. **(A)** Internal test set; **(B)** External Test Set; **(C)** Video Test Set.

Three endoscopists tested the internal and external test sets without being aware of the pathology results. The test results of GAM-EfficientNet and endoscopists are listed in **Table 3**. The ROC curves are shown in **Figure 5**. The area under the curve (AUC) of the GAM-EfficientNet was 98.79% (95% CI: 0.98 to 0.99) for the internal test set and 99.46% (95% CI: 0.99- 1.00) for the external test set. No overlap occurred between GAM-EfficientNet and the endoscopists. This indicated that AUC was statistically significantly different between GAM-EfficientNet and the endoscopists. The performance of GAM-EfficientNet in diagnosing CAG was significantly higher than that of the endoscopists.

**Table 3:** Diagnostic performance of GAM-EfficientNet and three endoscopists (n%).

DataSet	Diagnosed by	Sensitivity	Specificity	Precision	Accuracy	F1-score	AUC(95%CI)
Internal test set	Endoscopist1	88.70	85.33	85.98	87.02	87.32	87.01(95% CI:0.85-0.89)
	Endoscopist2	87.84	86.59	86.92	87.22	87.37	87.21(95% CI:0.86-0.89)
	Endoscopist3	89.85	87.66	88.08	88.76	88.95	88.75(95% CI:0.87-0.90)
	Endoscopists All	88.8 ± 0.82	86.53 ± 0.95	87 ± 0.86	87.67 ± 0.78	87.88 ± 0.76	87.66 ± 0.78
	GAM-EfficientNet	94.35	94.07	94.17	94.21	94.26	98.79(95% CI:0.98-0.99)
External test set	Endoscopist1	88.70	85.33	85.98	87.02	87.32	87.17(95% CI:0.84-0.90)
	Endoscopist2	87.84	86.59	86.92	87.22	87.37	85.67(95% CI:0.82-0.89)
	Endoscopist3	89.85	87.66	88.08	88.76	88.95	86.17(95% CI:0.83-0.9)
	Endoscopists All	87.22 ± 1.1	90.44 ± 0.42	90.12 ± 0.47	88.83 ± 0.72	88.65 ± 0.77	88.83 ± 0.72
	GAM-EfficientNet	93	94	93.94	93.5	93.47	99.46(95% CI:0.99-1.00)
Video test set	Endoscopist1	90.57	89.23	87.27	89.83	88.89	89.90(95% CI:0.84-0.96)
	Endoscopist2	90.57	87.69	85.71	88.98	88.07	89.72(95% CI:0.83-0.96)
	Endoscopist3	88.68	90.77	88.68	89.83	88.68	89.13(95% CI:0.83-0.96)
	Endoscopists All	89.94 ± 1.09	89.23 ± 1.54	87.22 ± 1.48	89.55 ± 0.49	88.55 ± 0.42	89.58 ± 0.4
	GAM-EfficientNet	96.23	89.23	87.93	92.37	91.89	92.73(95% CI:0.87-0.98)

We attempted to determine the reasons for these model prediction errors. In the internal test set, 120 images were incorrectly predicted, including 59 FN images from 48 patients and 61 FP images from 51 patients. Three endoscopists collaboratively diagnosed the 120 misidentified images. If endoscopists disagree on the diagnosis, they resolved through discussion. Of the 59 FN images, only four were diagnosed correctly. Nine of the 61 FP images were correctly identified. We found that it was difficult for experienced endoscopists to evaluate the images that the model incorrectly predicted. We analyzed the causes of these prediction errors. This study used pathology results as the gold standard and endoscopists selected the images in the dataset. However, the following two cases cannot be excluded: the endoscopist did not take the atrophy site when taking the pathology, and the image did not contain the area of pathology. In addition, different light intensities and angles could also affect the judgment of the model.

**Figure 6:**
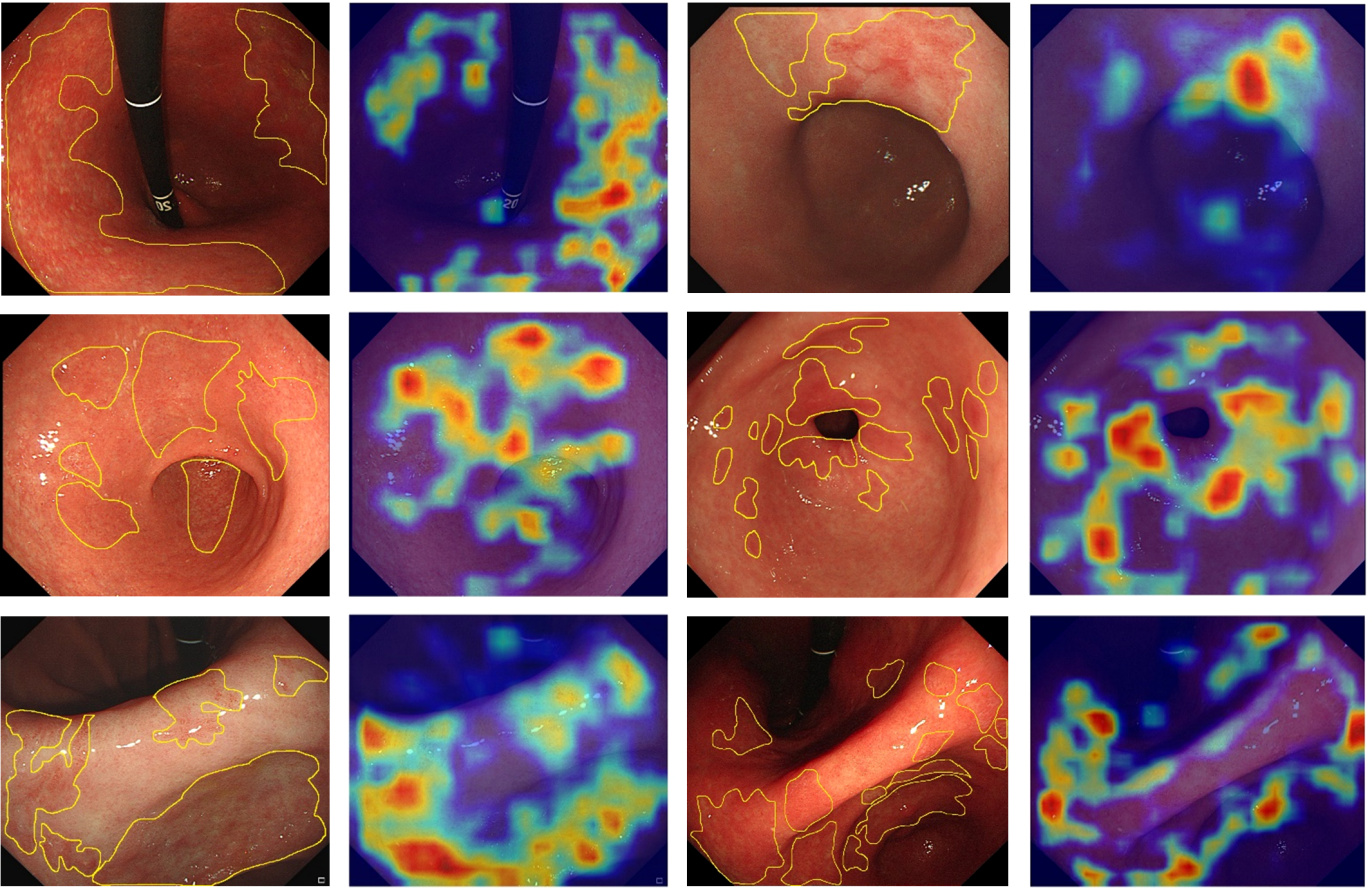
Feature heatmaps of the GAM-EfficientNet. To improve the quality of the heatmaps, we cropped the original endoscopic image and did not compress it.

The Grad-CAM heatmap highlights the regions of interest of the GAM-EfficientNet in red, yellow, and green. As shown in **Figure 6**, the endoscopists labeled some of the images with atrophied regions and compared them with the heatmaps generated by the model. The endoscopists’ annotations were generally consistent with the areas of concern for the model. In summary, GAM-EfficientNet can focus on meaningful regions in endoscopic images for CAG predictions. Heatmaps can also provide endoscopists with a visual basis for diagnosis.

### 3.4 Video verification

As the images used for model training, internal testing, and external testing were selected by the endoscopist, GAM-EfficientNet’s adaptation to more complex real-time endoscopic environments need further validation. To test the model, we collected video clips of 118 patients who underwent real-time endoscopy. A separate model was trained to recognize blurred frames in the videos. Blurred frames were ignored during the GAM-EfficientNet diagnosis. To prevent the model from misdiagnosis owing to one frame, we specified that five consecutive frames were diagnosed as CAG. Otherwise, the patient was diagnosed as having CNAG. Three endoscopists independently diagnosed the videos based on their experience. The diagnostic results of the model and endoscopists are listed in **Table 3**. **Supplementary Video 1** shows an example video of the model diagnostic process, which was converted to 10 fps to provide a better view of the diagnostic process. In the video test set, the F1-score and AUC of GAM-EfficientNet were 91.89% and 92.73%, respectively, still higher than those of the endoscopist.

## 4 Discussion

Gastric cancer is the fifth most prevalent type of cancer and the fourth most common cause of cancer-related deaths worldwide (1). Gastric mucosal atrophy is a critical stage in gastric cancer progression; the higher the degree of mucosal atrophy, the higher the risk of cancer (3). If we can improve the recognition of CAG and timely intervention, the incidence of gastric cancer and the mortality rate will be reduced. In recent years, DL technology has achieved considerable success in image recognition, and its application in assisted gastroscopy diagnosis is of great significance in improving disease recognition rates.

In this study, we created a new DL network, GAM-EfficientNet, based on EfficientNetV2-M by adding a GAM module. GAM introduces spatial and channel attention mechanisms that allow the network to focus more on valuable regions in the image. GAM-EfficientNet outperforms the other networks mentioned in the paper in terms of recognition ability, with sensitivity, specificity, and accuracy of 93%, 94%, and 93.5%, respectively, on the external test set. CAG is a precancerous disease that requires a high recall rate to reduce the number of missed diagnoses. The precision and recall rates of the GAM-EfficientNet were 93.94% and 93%, respectively, on the external test set, which suggests that the model has high precision and a low probability of missing diagnosis. The comprehensive evaluation indices F1-score and AUC were 93.47% and 99.46%, respectively, indicating the high value of the model as an aid to diagnosis. It provides a visual diagnostic basis for the endoscopist by generating a heatmap showing the areas of attention where the GAM-EfficientNet makes decisions.

To further validate the model’s performance, we tested it on a video test set and compared it with that of the three endoscopists. The results showed that the F1-score and AUC of GAM-EfficientNet were 91.89% and 92.73%, respectively, 1.58 and 6.73 percentage points lower than those on the external test set, respectively. The performance of all three endoscopists on the video test set improved, but remained lower than that of GAM-EfficientNet.

The model has limitations and scope for further improvement, notably the following: (1) Multi-center study. The training set images used in this study were obtained from one hospital, and they were high-quality images screened by endoscopists. However, the images may require more diversity. In a real environment, different devices and parameter settings can affect endoscopic image imaging, and factors such as the angle, light, food residue, and digestive fluid can affect the evaluation of the model. In the future, we will include multiple centers to collect more images of different models of endoscopic devices for training, to improve the generalization ability of the model. (2) The classification was further refined according to the severity of atrophy. In this study, we identified only CAG and did not distinguish its severity. The degree of atrophy may differ from the pathological findings at different positions in the same patient. In the future, we will collect endoscopic images strictly according to the locations where the pathological biopsy was conducted, and classify and train according to the pathological results showing the degree of atrophy, to improve the recognition effect of the model. (3) Labeling of atrophy sites. Our classification model only provides diagnostic results for the image, although the heatmap can provide some indications of the atrophy site. Ideally, the area of atrophy should be outlined precisely in the image, providing a more visual aid to the endoscopists. However, this is a challenging task. (4) The performance of AI may have been overestimated. All images in the experiment were selected by the endoscopists, which may have led to overfitting of the model. Although we used videos to simulate a real environment, the videos did not include other lesions. With the inclusion of other lesions, the recognition ability of the model requires further validation, which is one of our future works.

In this study, we constructed a deep learning-based CAG recognition model with higher diagnostic performance than that of endoscopists. This can provide an objective and reliable diagnostic basis for endoscopists.

## Data availability statement

The original contributions presented in the study are included in the article/Supplementary Material. Our code can be get from GitHub, URL: https://github.com/flyingfatpig/GAM-EfficientNet. Further inquiries can be directed to the corresponding authors.

## Ethics statement

This study was approved by the ethics committees of the two relevant hospitals involved (No. 202201016, Zibo Central Hospital. No. 202210019, Zhangdian Maternal and Child Health Hospital). Patients in the video test group provided written informed consent before participation. The ethics committee waived informed consent for patients who participated in retrospective imaging. Written informed consent was obtained from the individual(s) for the publication of any potentially identifiable images or data included in this article.

## Author contributions

YS and BL conceived the idea. BL built and trained the model. YS wrote the manuscript with support from the rest of the authors. NW, NL, and JW collected the data, analyzed the experimental results, and produced the figures. HW validated the experimental data. TT advised the project and revised the manuscript. All authors contributed to the article and approved the submitted version.

## References

[1] Sung H Ferlay J Siegel RL Laversanne M Soerjomataram I Jemal A. Global cancer statistics 2020: GLOBOCAN estimates of incidence and mortality worldwide for 36 cancers in 185 countries. CA Cancer J Clin (2021) 71:209–49. doi: 10.3322/caac.21660 33538338

[2] Correa P. A human model of gastric carcinogenesis. Cancer Res (1988) 48:3554–60. 3288329

[3] Banks M Graham D Jansen M Gotoda T Coda S di Pietro M. British Society of gastroenterology guidelines on the diagnosis and management of patients at risk of gastric adenocarcinoma. Gut (2019) 68:1545–75. doi: 10.1136/gutjnl-2018-318126 PMC6709778 31278206

[4] Weck MN Brenner H. Prevalence of chronic atrophic gastritis in different parts of the world. Cancer Epidemiol Biomarkers Prev (2006) 15:1083–94. doi: 10.1158/1055-9965.EPI-05-0931 16775164

[5] Asaka M Kato M Kudo M Katagiri M Nishikawa K Koshiyama H. Atrophic changes of gastric mucosa are caused by helicobacter pylori infection rather than aging: studies in asymptomatic Japanese adults. Helicobacter (1996) 1:52–6. doi: 10.1111/j.1523-5378.1996.tb00008.x 9398913

[6] You WC Zhang L Gail MH Li JY Chang YS Blot WJ. Precancerous lesions in two counties of China with contrasting gastric cancer risk. Int J Epidemiol (1998) 27:945–8. doi: 10.1093/ije/27.6.945 10024186

[7] Lahner E Zagari RM Zullo A Di Sabatino A Meggio A Cesaro P. Chronic atrophic gastritis: Natural history, diagnosis and therapeutic management. a position paper by the Italian society of hospital gastroenterologists and digestive endoscopists [AIGO], the Italian society of digestive endoscopy [SIED], the Italian society of gastroenterology [SIGE], and the Italian society of internal medicine [SIMI]. Digestive Liver Dis (2019) 51:1621–32. doi: 10.1016/j.dld.2019.09.016 31635944

[8] Eshmuratov A Nah JC Kim N Lee HS Lee HE Lee BH. The correlation of endoscopic and histological diagnosis of gastric atrophy. Dig Dis Sci (2010) 55:1364–75. doi: 10.1007/s10620-009-0891-4 19629687

[9] Kanawong R Obafemi-Ajayi T Ma T Xu D Li S Duan Y. Automated tongue feature extraction for ZHENG classification in traditional Chinese medicine. Evid Based Complement Alternat Med (2012) 2012:912852. doi: 10.1155/2012/912852 22693533 PMC3369473

[10] Gargeya R Leng T. Automated identification of diabetic retinopathy using deep learning. Ophthalmology (2017) 124:962–9. doi: 10.1016/j.ophtha.2017.02.008 28359545

[11] Choi E Schuetz A Stewart WF Sun J. Using recurrent neural network models for early detection of heart failure onset. J Am Med Inform Assoc (2017) 24:361–70. doi: 10.1093/jamia/ocw112 PMC5391725 27521897

[12] Medved D Ohlsson M Höglund P Andersson B Nugues P Nilsson J. Improving prediction of heart transplantation outcome using deep learning techniques. Sci Rep (2018) 8:3613. doi: 10.1038/s41598-018-21417-7 29483521 PMC5827028

[13] Helwan A Uzun Ozsahin D. Sliding window based machine learning system for the left ventricle localization in MR cardiac images. Appl Comput Intell Soft Computing (2017) 2017:e3048181. doi: 10.1155/2017/3048181

[14] Yan Z Zhan Y Peng Z Liao S Shinagawa Y Zhang S. Multi-instance deep learning: Discover discriminative local anatomies for bodypart recognition. IEEE Trans Med Imaging (2016) 35:1332–43. doi: 10.1109/TMI.2016.2524985 26863652

[15] Ueyama H Kato Y Akazawa Y Yatagai N Komori H Takeda T. Application of artificial intelligence using a convolutional neural network for diagnosis of early gastric cancer based on magnifying endoscopy with narrow-band imaging. J Gastroenterol Hepatol (2021) 36:482–9. doi: 10.1111/jgh.15190 PMC7984440 32681536

[16] Shichijo S Nomura S Aoyama K Nishikawa Y Miura M Shinagawa T. Application of convolutional neural networks in the diagnosis of helicobacter pylori infection based on endoscopic images. EBioMedicine (2017) 25:106–11. doi: 10.1016/j.ebiom.2017.10.014 PMC5704071 29056541

[17] LeCun Y Boser B Denker JS Henderson D Howard RE Hubbard W. Backpropagation applied to handwritten zip code recognition. Neural Comput (1989) 1:541–51. doi: 10.1162/neco.1989.1.4.541

[18] Krizhevsky A Sutskever I Hinton GE. ImageNet classification with deep convolutional neural networks. Commun ACM (2017) 60:84–90. doi: 10.1145/3065386

[19] Zhao S-B Yang W Wang S-L Pan P Wang R-D Chang X. Establishment and validation of a computer-assisted colonic polyp localization system based on deep learning. WJG (2021) 27:5232–46. doi: 10.3748/wjg.v27.i31.5232 PMC8384745 34497447

[20] Guimarães P Keller A Fehlmann T Lammert F Casper M. Deep-learning based detection of gastric precancerous conditions. Gut (2020) 69:4–6. doi: 10.1136/gutjnl-2019-319347 31375599

[21] Mu G Zhu Y Niu Z Li H Wu L Wang J. Expert-level classification of gastritis by endoscopy using deep learning: a multicenter diagnostic trial. Endosc Int Open (2021) 9:E955–64. doi: 10.1055/a-1372-2789 PMC8159578 34079883

[22] Tytgat GN. The Sydney system: endoscopic division. endoscopic appearances in gastritis/duodenitis. J Gastroenterol Hepatol (1991) 6:223–34. doi: 10.1111/j.1440-1746.1991.tb01469.x 1912432

[23] Fang J-Y Du YQ Liu WZ Ren JL Li YQ Chen XY. Chinese Society of gastroenterology, Chinese medical association. Chinese consensus on chronic gastritis (2017, shanghai). J Dig Dis (2018) 19:182–203. doi: 10.1111/1751-2980.12593 29573173

[24] Vaswani A Shazeer N Parmar N Uszkoreit J Jones L Gomez AN. Attention is all you need. In Proceedings of the 31st International Conference on Neural Information Processing Systems. NIPS’17. Red Hook, NY, USA: Curran Associates Inc. (2017). p. 6000–10.

[25] Dosovitskiy A Beyer L Kolesnikov A Weissenborn D Zhai X Unterthiner T. An image is worth 16x16 words: Transformers for image recognition at scale. arXiv [Preprint]. (2021). doi: 10.48550/arXiv.2010.11929

[26] Liu Z Lin Y Cao Y Hu H Wei Y Zhang Z. Swin transformer: Hierarchical vision transformer using shifted windows. In 2021 IEEE/CVF International Conference on Computer Vision (ICCV) (2021), 9992–10002. doi: 10.1109/ICCV48922.2021.00986

[27] Li S Sui X Luo X Xu X Liu Y Goh R. Medical image segmentation using squeeze-and-Expansion transformers. In Proceedings of the Thirtieth International Joint Conference on Artificial Intelligence. Montreal, Canada: International Joint Conferences on Artificial Intelligence Organization (2021) p. 807–815. doi: 10.24963/ijcai.2021/112

[28] Xie E Wang W Wang W Sun P Xu H Liang D. Segmenting Transparent Objects in the Wild with Transformer. In Proceedings of the Thirtieth International Joint Conference on Artificial Intelligence. Montreal, Canada: International Joint Conferences on Artificial Intelligence Organization (2021) p. 1194–1200. doi: 10.24963/ijcai.2021/165

[29] Ren S He K Girshick R Sun J.Faster R-CNN: Towards Real-Time Object Detection with Region Proposal Networks. IEEE Trans Pattern Anal Mach Intell (2017) 39:1137–1149. doi: 10.1109/TPAMI.2016.2577031 27295650

[30] Lin T-Y Goyal P Girshick R He K Dollar P. Focal Loss for Dense Object Detection. In 2017 IEEE International Conference on Computer Vision (ICCV).Venice: IEEE (2017) p. 2999–3007. doi: 10.1109/ICCV.2017.324

[31] Tan M Le Q. EfficientNetV2: Smaller models and faster training. In Proceedings of the 38th International Conference on Machine Learning. PMLR (2021) p. 10096–10106.

[32] Liu Z Mao H Wu C-Y Feichtenhofer C Darrell T Xie S. A ConvNet for the 2020s. In 2022 IEEE/CVF Conference on Computer Vision and Pattern Recognition (CVPR).New Orleans, LA, USA: IEEE (2022) p. 11966–11976. doi: 10.1109/CVPR52688.2022.01167

[33] Deng J Dong W Socher R Li L-J Kai L Li F-F. ImageNet: A large-scale hierarchical image database, in: IEEE Conference on Computer Vision and Pattern Recognition. Miami, FL: IEEE (2009). pp. 248–55. doi: 10.1109/CVPR.2009.5206848

[34] Russakovsky O Deng J Su H Krause J Satheesh S Ma S. ImageNet Large scale visual recognition challenge. Int J Comput Vis (2015) 115:211–52. doi: 10.1007/s11263-015-0816-y

[35] Pan SJ Yang Q. A survey on transfer learning. IEEE Trans Knowl Data Eng (2010) 22:1345–59. doi: 10.1109/TKDE.2009.191

[36] Day O Khoshgoftaar TM. A survey on heterogeneous transfer learning. J Big Data (2017) 4:29. doi: 10.1186/s40537-017-0089-0

[37] Liang G Zheng L. A transfer learning method with deep residual network for pediatric pneumonia diagnosis. Comput Methods Programs BioMed (2020) 187:104964. doi: 10.1016/j.cmpb.2019.06.023 31262537

[38] Huang C Lv W Zhou C Mao L Xu Q Li X. Discrimination between transient and persistent subsolid pulmonary nodules on baseline CT using deep transfer learning. Eur Radiol (2020) 30:6913–23. doi: 10.1007/s00330-020-07071-6 32696253

[39] Ridnik T Ben-Baruch E Noy A Zelnik-Manor L. ImageNet-21K pretraining for the masses. arXiv [Preprint] (2021). doi: 10.48550/arXiv.2104.10972

[40] Cao R. Towards Accelerated and Robust Rreinforcement Learning with Transfer Learning. In 2022 International Conference on Big Data, Information and Computer Network (BDICN). Sanya, China: IEEE (2022). p. 335–40. doi: 10.1109/BDICN55575.2022.00069

[41] Liu Y Shao Z Hoffmann N. Global attention mechanism: Retain information to enhance channel-spatial interactions. arXiv[Preprint] (2021). doi: 10.48550/arXiv.2112.05561

[42] He T Zhang Z Zhang H Zhang Z Xie J Li M. Bag of Tricks for Image Classification with Convolutional Neural Networks. In 2019 IEEE/CVF Conference on Computer Vision and Pattern Recognition (CVPR). Long Beach, CA, USA: IEEE (2019). p. 558–67. doi: 10.1109/CVPR.2019.00065

[43] Aggarwal R Sounderajah V Martin G Ting DSW Karthikesalingam A King D. Diagnostic accuracy of deep learning in medical imaging: a systematic review and meta-analysis. NPJ Digit Med (2021) 4:65. doi: 10.1038/s41746-021-00438-z 33828217 PMC8027892

[44] Haenssle HA Fink C Schneiderbauer R Toberer F Buhl T Blum A. Man against machine: diagnostic performance of a deep learning convolutional neural network for dermoscopic melanoma recognition in comparison to 58 dermatologists. Ann Oncol (2018) 29:1836–42. doi: 10.1093/annonc/mdy166 29846502

[45] Sollini M Antunovic L Chiti A Kirienko M. Towards clinical application of image mining: a systematic review on artificial intelligence and radiomics. Eur J Nucl Med Mol Imaging (2019) 46:2656–72. doi: 10.1007/s00259-019-04372-x PMC6879445 31214791

[46] Barredo Arrieta A Díaz-Rodríguez N Del Ser J Bennetot A Tabik S Barbado A. Explainable artificial intelligence (XAI): Concepts, taxonomies, opportunities and challenges toward responsible AI. Inf Fusion (2020) 58:82–115. doi: 10.1016/j.inffus.2019.12.012

[47] Tjoa E Guan C. A survey on explainable artificial intelligence (XAI): Toward medical XAI. IEEE Trans Neural Netw Learn Syst (2021) 32:4793–813. doi: 10.1109/TNNLS.2020.3027314 33079674

